# The Road to Dog Rabies Control and Elimination—What Keeps Us from Moving Faster?

**DOI:** 10.3389/fpubh.2017.00103

**Published:** 2017-05-15

**Authors:** Anna S. Fahrion, Louise H. Taylor, Gregorio Torres, Thomas Müller, Salome Dürr, Lea Knopf, Katinka de Balogh, Louis H. Nel, Mary Joy Gordoncillo, Bernadette Abela-Ridder

**Affiliations:** ^1^Neglected Zoonotic Diseases, Department of Control of Neglected Tropical Diseases, World Health Organization, Geneva, Switzerland; ^2^Global Alliance for Rabies Control, Manhattan, KS, USA; ^3^World Organisation for Animal Health, Paris, France; ^4^Institute of Epidemiology, Friedrich-Löffler-Institut, Federal Research Institute for Animal Health, Greifswald, Germany; ^5^Veterinary Public Health Institute, University of Bern, Bern, Switzerland; ^6^Food and Agriculture Organization of the United Nations, Bangkok, Thailand; ^7^Department of Microbiology and Plant Pathology, Natural and Agricultural Sciences, University of Pretoria, Pretoria, South Africa

**Keywords:** rabies, dog rabies, neglected tropical diseases, zero human deaths, global framework, implementation

## Abstract

Rabies, a vaccine preventable neglected tropical disease, still claims an estimated 35,000–60,000 human lives annually. The international community, with more than 100 endemic countries, has set a global target of 0 human deaths from dog-transmitted rabies by 2030. While it has been proven in several countries and regions that elimination of rabies as a public health problem is feasible and tools are available, rabies deaths globally have not yet been prevented effectively. While there has been extensive rabies research, specific areas of implementation for control and elimination have not been sufficiently addressed. This article highlights some of the commonest perceived barriers for countries to implementing rabies control and elimination programs and discusses possible solutions for sociopolitical, organizational, technical, and resource-linked requirements, following the pillars of the global framework for the elimination of dog-mediated human rabies adopted at the global rabies meeting in December 2015.

## Background

During the past decade, neglected tropical diseases (NTDs) have gained more recognition on the global health and development agendas (1–3). The transition from the Millennium Development Goals to the Sustainable Development Goals has renewed emphasis on ending the inequality that has deprived neglected communities from access to effective and affordable health care (Goal 3.8) and includes a specific goal to end NTDs by 2030 (Goal 3.3) (4).

Rabies, a viral disease categorized by the World Health Organization (WHO) as a NTD, kills tens of thousands of people every year, mostly among underserved populations in Africa and Asia; more than 95% of human rabies deaths result from the bites of infected dogs (5, 6). While the disease is almost 100% fatal, effective human and dog vaccines to prevent rabies are available. Elimination of dog-transmitted rabies as a public health problem is feasible (7, 8) by vaccinating dogs and providing post-exposure prophylaxis (PEP) to humans until dog rabies is eliminated (5).

**Figure 1:**
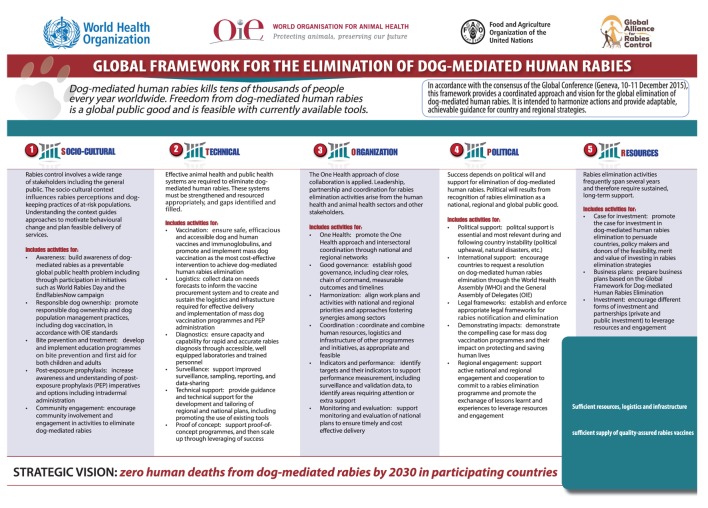
**Global framework for the elimination of dog-mediated human rabies**.

Elimination of canine rabies is integral to the WHO–OIE^1^–FAO^2^ tripartite collaboration, which works at the animal–human–ecosystems interface (9, 10). A joint global meeting (Geneva, December 2015) marked a milestone at which human and animal health sectors agreed a framework to eliminate canine rabies with the vision of ending dog-mediated human rabies by 2030 (8) (Figure 1). All 180 Member countries of the OIE affirmed this commitment in Resolution N.26 adopted by the World Assembly of Delegates of the OIE in May 2016.^3^

Rabies is widely recognized as a public health threat that warrants prioritization of control efforts in Asia (11–13), Africa (14), and among the least developed nations globally (15). Health leaders are increasingly aware that this fatal disease could be eliminated as a public health problem cost effectively in a relatively short time (7, 16), yet rabies remains neglected and progress remains slow.

While there has been extensive research on the rabies virus, a comparative lack of operational research has led to knowledge gaps in how to design and implement control and elimination programs where they are needed most (11, 17) and calls for a “science of rabies elimination” (18). What barriers remain to coordinated efforts within and among countries? What is needed to transform the increased public and political awareness into real progress on the ground? And ultimately, what is needed to translate existing knowledge into success against rabies in a country? This paper discusses aspects of the “science to policy gap,” (perceived) barriers to progress and possible solutions (Table 1). It is structured according to the pillars of the Global framework for the elimination of dog-mediated human rabies (Figure 1), namely, sociocultural, technical, organization, political, and resources, reflecting a coordinated approach.

**Table 1:** **Key areas for improvement, necessary actions, and the stakeholders required to take action to support programmatic success for canine rabies elimination**.

Pillar	Action	By who	Main target audiences/beneficiaries
**Potential barrier: lack of awareness and prioritization**			

Political	Demonstrate the burden and impact	Epidemiologists, public health and veterinary services, program managers, international organizations^a^	Government policy makers, global health funders

Political	Declare the disease notifiable	Government lawmakers, World Health Organization (WHO), OIE	Health and veterinary professionals

Political	Implement adequate surveillance in both humans and animals	Policy makers, public health and veterinary authorities	Local authorities, health and veterinary professionals

Sociocultural	Build awareness of the risks and prevention methods	All stakeholders, but especially: health educators, media, program managers, international organizations^a^	General public, particularly children

Sociocultural	Build community engagement and responsible dog ownership	Policy makers, health communicators, communities, NGOs	Communities/general public, dog owners

**Potential barrier: lack of necessary guidance at regional and national level**			

Organizational	Plan effective interventions	Program managers with support of international organizations^a^ and experienced countries with successful programs as examples	National implementation authorities

Organizational	Enable intersectoral collaboration at local and national levels	All relevant government sectors, NGOs and private partners, international organizations^a^	Program managers and health-care providers

Organizational	Regional collaboration	Regional networks and (economic) associations, direct country partnerships, international organizations^a^	Program managers

**Potential barrier: cumbersome methodologies**			

Technical	Simplify rabies diagnosis for surveillance	Researchers, test developers and producers	National and regional laboratory and surveillance personnel

Technical	Simplify access to vaccine	(Regional or national) responsibilities for procurement mechanisms, OIE and WHO vaccine banks	Government policy makers, global health funders, Program managers

Technical	Simplify vaccine regimen and delivery	Researchers, expert groups developing recommendations^b^ and guidelines, logisticians	Health authorities, community health providers

Technical	Improve effectiveness of vaccination strategies	Program managers, epidemiologists/researchers	Program implementers

Technical	Assess and implement control and management of dog movement	Policy makers, veterinary authorities, researchers	Local authorities, dog owners

**Potential barrier: inadequate funding**			

Resources	Ensure adequate resources for a program	Governments and international and bilateral funding agencies [including (international) funding agencies, foundations, private donors/investors, etc.]	Government and local policy makers, global health funders, program managers

Resources	Build necessary capacity and expertise for sustained control	National capacity building agencies, international organizations^a^	Government health departments, local authorities, medical and veterinary officers

Resources	Build a business plan for global rabies elimination	WHO, OIE, FAO, GARC	Government policy makers, (global) health funders, program managers

*^a^“International organizations” refers primarily to the FAO/OIE/WHO tripartite and global NGOs such as Global Alliance for Rabies Control (GARC). But these roles and principles are equally valid for other organizations working in rabies or zoonosis control.^b^A WHO Strategic Advisory Group of Experts on Immunization (SAGE) working group on rabies vaccines and rabies immunoglobulins was established in 2016 and is currently reviewing the scientific evidence and relevant programmatic considerations on the use and scheduling of these. The proposed recommendations resulting from this work will be considered by SAGE during its October 2017 meeting*.

## Overcoming Barriers to Rabies Elimination

### Political or Sociocultural—Raising Public Awareness and Political Will

#### Making the Burden Visible, Demonstrating Impact

Prioritization of a disease is brought about through increasing public awareness and political will (19). One of the most important means of convincing policy makers to prioritize a disease and invest resources is to demonstrate its impact on public health and the economy and the potential benefit of targeting the disease.

High-quality surveillance data are needed, but human rabies deaths are commonly underreported 100-fold (20–22), and the absence of data and solid evidence for estimates induces a cycle of neglect (23). Conversely, the onset of a control program that delivers better surveillance data is a precondition to increased awareness. Where quantitative assessments have been attempted, rabies has been ranked consistently among the top five zoonotic diseases, for example, in India (12), Mongolia (13), Jordan (24), Ethiopia (25), Myanmar (26), and Kenya (27).

Declaring a disease notifiable is crucial to establish functional reporting (28), and monitoring and surveillance of the disease should, therefore, be a central element of every rabies program. Rabies is also included in the OIE list of notifiable diseases.^4^ Disease surveillance starts at the community level, where awareness about the disease needs to be complemented by clear guidance on reporting to the authorities, ideally integrated into the wider national health information and statistics systems. Pathways must be included for transmission of data from the community level to the national level and to the OIE and WHO, resulting in feedback and action to keep individuals along the reporting chain informed and engaged. To ensure that data are comparable and informative, indicators should be well-defined and measurable. Novel technology such as notification *via* cell phones could be further explored (29).

#### Creating Public Awareness

Rabies burdens individuals, families, societies, and economies (6). As communities become aware of this threat, political pressure to act will accumulate. Building awareness and education about how to avoid and treat rabies exposures is, therefore, crucial in mobilizing a country to eliminate rabies. Champions at all levels (community to national) are central to this effort as they directly advocate and educate communities (7). World Rabies Day, recognized by the United Nations and commemorated every year on 28th September, celebrated its 10th anniversary in 2016 with 302 events in 57 countries (as of December 21, 2016). This annual awareness-raising event has shown a remarkable upwards trend since its inception and is an example of the dedication of innumerable people worldwide (30, 31).

#### Building an Engaged Society

An integral part of a regional or national plan is to build a proactive society that is fully engaged in the dog rabies elimination efforts of the country. Awareness of rabies at the community level alone is not enough to increase pressure on governments to improve their control efforts. Besides well-informed general public and responsible pet owners, committed, supportive policy makers are needed who will cohesively support national efforts to achieve and maintain freedom from rabies. Currently, most efforts to raise public awareness focus on promoting rabies information, which may not translate into the desired behavior, practices, and actions. Thus, it is important to invest in a national communication strategy and in impact monitoring that use the science of behavioral change and consider the diverse behavioral drivers, incentives, motivations, and larger sociocultural context of the target audience.

An example of behavioral change necessary for rabies control is that owners accept responsibility for their dogs and any offspring they may produce. This includes protecting dogs from rabies through vaccination and from unwanted reproduction. The promotion of such responsible dog ownership can be achieved only through a combination of adequate legislation, public awareness, and education, recognizing cultural and economic conditions. Public health and veterinary authorities, animal welfare organizations, and private veterinarians should work together to establish and maintain responsible dog ownership programs especially in communities at risk.

### Organizational—Establishing Necessary Policies and Guidance

#### Effective Planning of Elimination Programs

National authorities are responsible for developing national strategies and implementing programs but they are frequently overwhelmed by multiple human and animal disease priorities and the challenges associated with programs stretched across sectors and administrative levels. It may be difficult to know where to start and what is needed—a potential barrier. Guidance for developing and monitoring control and elimination programs is, however, freely available. For example, the Stepwise Approach toward Rabies Elimination, which is embedded in the rabies blueprint,^5^ follows the principles of enhancing intersectoral collaboration. This guidance has been used by countries across three continents, mostly at national or regional stakeholder consultations, to kick-start coordinated rabies control (32). Likewise, the rabies surveillance blueprint^6^ provides guidance for planning of surveillance in particular. Knowledge about these tools needs to be disseminated and promoted more widely.

#### Intersectoral Collaboration

While the incremental benefits of a One Health approach for rabies control are established at the highest international level, its operationalization at national or local levels remains a challenge. Administrative and management structures may need to be harmonized across sectors according to different ministries and budget lines and coordinated with stakeholders from the private sector (33). National stakeholder consultations that convene all actors across ministries, local and national levels as well as the private and public sectors, however, have proven excellent platforms from which to build connections and trust and from where operational barriers and constraints to effective collaboration in rabies prevention as well as possible solutions can be explored. The outcome of these consultations is the drafting of integrated, multidisciplinary rabies action plans (34). For example, coordination at the national level can pave the way for integrated management of bite cases at the local level, jointly involving human and animal professionals to ensure reporting of bite and rabies incidents, proper risk assessments, and a coordinated response while at the same time sharing logistic resources (35, 36). Close involvement of social sciences, the education sector, and municipalities is now equally recognized, for example, as a powerful method for preventing dog bites in children, increasing knowledge and awareness about rabies, and in sustainably managing dog populations in affected communities (36).

#### Sharing and Comparing: Transparency and Regional Collaboration

International cooperation and coordination in planning, implementing, and evaluating rabies control programs at all levels is crucial for success and cost effectiveness (5). As canine rabies is a transboundary disease, collaboration, cooperation, and transparency between countries can provide new insights for tackling the disease. Authorities often perceive admitting public health problems as failure and prefer not to address endemic rabies at international level, thereby missing an opportunity to share information. Establishing contacts between public health and veterinary authorities of neighboring regions and countries and frequently exchanging information and data can be a first step toward building trust and more regular bilateral or multilateral interaction. WHO collaborating centers, OIE, and FAO reference laboratories and other organizations can help countries to share, compare, and learn from each other’s experience (5, 37). Participation at international disease conferences can also attract more international attention at a political level.

A regional approach has been fundamentally important for the effective control of rabies in Western Europe (5) and, more recently, Latin America (38). Rabies control program managers from across Latin America and the Caribbean meet at meetings facilitated by the Pan American Health Organization (REDIPRA) and the large scale, parallel declines in dog and human rabies achieved are striking (39). In Asia, the Association of Southeast Asian Nations launched a joint Rabies Elimination Strategy that encapsulates a regional approach in 2015.^7^ The Middle Eastern, Eastern Europe, Central Asia, and North Africa Rabies Experts Bureau has held meetings since 2010 (40) and has called for a strong regional initiative with high level political support (41). In sub-Saharan Africa, the Pan-African Rabies Control Network was created in 2015 and shows promise as a suitable platform to drive a regional approach to rabies control (37). Such regional approaches will be vital to implementing the global strategic framework to eliminate dog-mediated rabies and will, therefore, require further extension with strong participation and political support from all countries for success.

### Technical—Ensuring Necessary Technology and Knowledge

#### Diagnostics

Diagnostic tests confirm animal rabies cases, allowing better PEP decision-making and monitoring the progress of control efforts. The reference, fluorescent antibody test, is not practicable in many endemic settings due to costs and enhanced laboratory requirements. Thus, alternative tools using less specialized equipment, such as Direct Rapid Immunohistochemical Test (42) and lateral flow devices (43), could play an important role provided further validation and quality approval.

#### Predicting the Need for Vaccine: Forecasting

Manufacturing cycles of both human and animal rabies vaccines require several years of appropriate forecasting by countries in order to supply the actual need. An absence of accurate data impairs forecasting and thereby resource allocation. The veterinary services of endemic countries often have insufficient knowledge of dog population size or ecology and human health services lack accurate data on bite case exposures. Both sectors thus suffer from procurement delays or stock shortages, which can result in less effective control of the disease and may force countries to turn to manufacturers selling vaccines that are overly expensive or may not meet international quality standards (44). Vaccine banks or stockpiles at regional levels as managed by OIE or WHO have become a solid mechanism for countries to maintain the supply of quality-assured vaccines and allow manufacturers to forecast and stabilize their production over years with lowered pricing through bulk purchase (45). Moreover, vaccine banks have contributed demonstrably to the scaling up and maintenance of local, national, or sub-regional programs in Asia and Africa (46) and incentivized recipient countries to increase data collection, as reporting on vaccine use and results is required. The opportunity of a potential investment from GAVI (the Vaccine Alliance) into human rabies vaccine from 2018 onward (47) could substantially facilitate low-income countries’ access to affordable rabies vaccine and stimulate the necessary political will to tackle human rabies at a large scale.

#### Getting the Vaccines to the Community

Despite the encouraging improvements observed in the field of universal health coverage, poor accessibility to and affordability of PEP [particularly of rabies immunoglobulin (RIG)] remain in most rabies-endemic countries (48). Certainly, progress has been made, for example, on shortening PEP regimens (fewer health facility visits) and countries changing their policy to cost-saving intradermal administration of rabies vaccines as recommended by WHO (49). There is hope that new technologies currently under evaluation by WHO (thermostable rabies vaccine, monoclonal antibodies as an alternative to human and equine RIG) will facilitate cost-effective delivery of PEP as well as dog vaccine to where it is needed. The long needed scale up and adaption of mechanisms for supply and distribution of PEP has received a global push through discussions around a potential GAVI investment.

#### More Tailored Dog Vaccination Strategies

Rabies is integrally linked to the ways people live with their dogs. Its control requires an adequate understanding of the dog ecology and dog-keeping practices in a country in locally differing sociocultural contexts (e.g., urban vs rural, among different economic, religious, or ethnic groups). Factors that can profoundly affect rabies transmission and control are usually not sufficiently understood to design the most appropriate control strategy, and as a result, efforts and resources can be wasted. In most circumstances, almost all dogs can be handled and vaccinated by the parenteral route (50). In rare cases, however, dogs may not be accessible to parenteral vaccination, thus jeopardizing the coverage of vaccination campaigns. Improved dog capture and vaccination techniques, as shown through the establishment of rabies A-teams by FAO in Bali, can assist with reaching dogs that are difficult to capture and handle.^8^ Oral dog vaccination using a hand-out model may be used effectively to immunize those inaccessible dogs while ensuring the safety of the vaccinators, the community, and non-target species. The best feasible solution for long-lasting marking of vaccinated dogs should be decided on during planning of campaigns (51).

The lack in dog movement control has been attributed as responsible for rabies spread in endemic areas and incursion in previously free countries or regions. Vaccinating at least 70% of animals in order to eliminate rabies from a free-roaming dog population is a widely acknowledged recommendation (8). However, empirical work [e.g., on area-specific basic reproductive ratio (*R*_0_) (52)] suggests that different settings probably require different vaccination strategies and coverages to control rabies successfully. High vaccination coverage in high-risk areas may be more crucial than medium coverage across the whole country, but clear guidance on this is lacking. Better knowledge of area/country specific factors related to dog-keeping practices, dog population turnover, and contact rates between dogs and wildlife can help in determining a more flexible, realistic required dog vaccination coverage. This can help to optimize resource allocation (53, 54) and define the most appropriate vaccination strategies and financing (55), the best vaccination campaign frequency (52), and how to target vaccination to the highest risk areas and segments of the dog population.

### Making Resources Available

As for any public health program, sustainable funding sources are a precondition to starting a rabies control program, and the absence of those sources is one of the main barriers. There are ways, however, to reduce the investments necessary for rabies control and to integrate rabies into existing streams of work and financing.

Recognizing rabies control as a public good and thus as the responsibility of national governments is key to a sustainable rabies elimination effort. Donor contributions should be structured so as to be catalytic to the establishment of the program, and long-term dependence on donor support should be avoided through a well-planned strategy for donor exit.

In most rabies-endemic countries, human PEP and vaccinating animals against rabies remain an “out-of-pocket” market. As they build on their commitments to universal health coverage and the sustainable development goals (56), governments need to step up their funding and integrate rabies control into sustainable health plans, ensuring dog vaccination, and PEP are available at the primary health-care level. Costs can be substantially decreased by using the intradermal route for human vaccines and tailoring dog vaccination to local circumstances (see above). Rabies surveillance and control in dogs should be integrated into existing infectious diseases reporting mechanisms and vaccination programs, requiring also more integration of funding streams. For example, in the Philippines, the ministry of health invests in dog vaccination as a public health measure, creating a leverage effect (57). Innovative mechanisms for financing and cost sharing can help to make vaccine purchase more affordable. To support funding bodies in planning and costing, WHO is currently developing in collaboration with FAO, OIE, and the Global Alliance for Rabies Control, a comprehensive business plan that encompasses both human and animal perspectives for achieving the 2030 target. In the longer term, investing in rabies prevention and control is cost-efficient, saving both lives and money (16, 58). It is possible to start small and then unlock more investment by demonstrating value.

Capacity building is an investment in human resources with crucial importance for the sustainability of a project. Parallel to the technical efforts made by the country, plans to build related capacity through training, professional development, and/or continuing education will ensure quality at implementation and ensure future sustainability. This contribution to health systems’ strengthening can be a legacy that rabies control efforts leave to a nation.

## Conclusion

This paper describes some of the main technical, organizational, and political challenges that countries encounter when implementing measures to control and eliminate rabies. However, there are solutions to many of these perceived barriers and opportunities to fill existing gaps. As summarized in Table 1, health officials, program managers, donors/investors, and all those involved in development of strategies should be aware of those applicable to their local, national, and regional contexts as early as possible, to make coordinated, informed decisions and successfully fight this devastating disease.

## Author Contributions

BA-R, LT, and AF prepared the initial concept for this paper. All the authors contributed equally to the further development of the content, delivered specific paragraphs for the paper from their area of expertise, and reviewed and complemented the manuscript. AF coordinated and collated the coauthor’s input.

## Conflict of Interest Statement

The authors declare an absence of any commercial or financial relationships to the content of the paper that could constitute a potential conflict of interest. The reviewer ENJ and handling editor declared their shared affiliation, and the handling editor states that the process nevertheless met the standards of a fair and objective review.

## Footnotes

^1^World Organization for Animal Health.

^2^Food and Agriculture Organization of the United Nations.

^3^http://www.oie.int/fileadmin/Home/eng/About_us/docs/pdf/Session/2016/A_RESO_2016_public.pdf.

^4^http://www.oie.int/en/international-standard-setting/terrestrial-code/access-online/.

^5^http://caninerabiesblueprint.org/.

^6^http://rabiessurveillanceblueprint.org/.

^7^http://vncdc.gov.vn/files/article_attachment/2015/3/endorsed-ares-final.pdf.

^8^http://www.fao.org/indonesia/news/detail-events/en/c/411271/.
